# Prevalence-Guided Anti-HCV and Reflex HCV Ag Testing in the Detection of Patients with Chronic Hepatitis C in Hepatitis C Endemic Areas

**DOI:** 10.3390/diagnostics15233064

**Published:** 2025-12-01

**Authors:** Sheng-Hsueh Chen, Yuan-Jie Ding, Nien-Tzu Hsu, Te-Sheng Chang, Yu-Chen Lin, Wen-Hua Chao, Sheng-Nan Lu

**Affiliations:** 1 Medical Education Committee, Chang Gung Memorial Hospital, Kaohsiung 833, Taiwan; 2 School of Medicine, Chang Gung University, Taoyuan 333, Taiwan; 3 Division of Gastroenterology and Hepatology, Department of Internal Medicine, Chang Gung Memorial Hospital, Chiayi 613, Taiwan; 4 Division of Gastroenterology and Hepatology, Department of Internal Medicine, Jen-Ai Hospital—Dali Branch, Taichung 41265, Taiwan; 5 Biostatistics and Bioinformatics Center, Kaohsiung Chang Gung Memorial Hospital, Kaohsiung 833, Taiwan; 6 Public Health Bureau, Chiayi 61249, Taiwan; 7 Division of Hepato-Gastroenterology, Department of Internal Medicine, Kaohsiung Chang Gung Memorial Hospital, Kaohsiung 833, Taiwan

**Keywords:** hepatitis C virus, reflex HCV antigen testing, community-based screening, village-level prevalence mapping, linkage-to-care

## Abstract

**Background/Objectives**: Chronic hepatitis C virus (HCV) remains a major public health concern in Taiwan, particularly in southern regions with high endemicity. While HCV elimination is a national priority, resources are often limited. Relying solely on broad, township-level prevalence rates is inefficient, as the true disease burden can vary dramatically at the village level. Therefore, identifying local hotspots through fine-scale mapping is critical for efficient resource allocation and targeted intervention. This study aimed to validate village-level prevalence estimates and evaluate the efficiency of a community-based, targeted screening approach utilizing this detailed prevalence data in Chiayi County. **Methods**: We integrated data from the Chiayi Health Bureau and Chiayi Chang Gung Memorial Hospital (2000–2015) to generate village-level risk maps for five townships: Lioujiao (LJ), Yijhu (YH), Dongshih (DS), Taibao (TB), and Lucao (LC). Between 2018 and 2021, we conducted door-to-door community screening using anti-HCV testing with reflex HCV antigen (Ag) testing. Anti-HCV/HCV Ag prevalence, number needed to test (NNT), and linkage-to-care rates were calculated to validate prevalence estimates and assess screening efficiency. **Results**: Among 3910 participants, anti-HCV prevalence ranged from 5.4% (TB) to 8.7% (DS). Estimated and observed village-level prevalence showed moderate-to-strong correlation (r = 0.696–0.830, *p* < 0.001). Screening efficiency was highest in DS (NNT = 21) and lowest in TB (NNT = 42). Of 132 antigen-positive individuals, 131 (99.2%) initiated direct-acting antiviral therapy. **Conclusions**: The village-level risk maps accurately predicted local HCV burden, enabling targeted screening with high diagnostic yield and near-complete treatment uptake. This approach maximizes resource efficiency and may serve as a scalable model for advancing Taiwan and the WHO’s 2030 HCV elimination goals.

## 1. Introduction

Chronic hepatitis C virus (HCV) infection has long posed a significant global public health burden. Without timely diagnosis and treatment, acute HCV infection often progresses to chronic infection, leading to liver fibrosis, cirrhosis, and potentially hepatocellular carcinoma (HCC) or liver failure. These outcomes are associated with substantial morbidity, mortality, and a marked decline in patients’ quality of life [1].

According to the World Health Organization (WHO), an estimated 71 million people were living with chronic HCV globally in 2015, with 1.75 million new infections that year [2]. In response, the WHO set global elimination targets in 2016 and released country-level validation guidance in 2023 [3]. These targets include diagnosing 90% of chronic HCV cases and recommending routine screening in populations with a prevalence over 5% [4]. Taiwan’s strategy aligns with these goals, aiming to reduce new infections by 90%, hepatitis-related deaths by 65%, and expand access to diagnosis and treatment [5].

In Taiwan, the prevalence of HCV has historically exceeded global averages, with particularly high endemicity in southern regions of Taiwan, including Chiayi, Yunlin, and Tainan [6]. Understanding such regional distributions is essential for the implementation of effective screening and treatment strategies. Although a nationwide township-specific risk map of high prevalence of anti-HCV has been published, a screening in Tainan County showed that marked geographic heterogeneity of anti-HCV prevalence was observed in smaller geographic units, specifically from 0% to 90.5% across 533 villages [7].

While HCV elimination is a global priority, the epidemiological challenges and diagnostic strategies vary significantly across different regions. For instance, the complexity of HCV epidemiology is often first seen in the variations among different risk groups, as illustrated by studies in the European context showing changes in prevalence and transmission routes between specific groups and the general population [8]. Furthermore, this heterogeneity extends to geographic distribution, where the need for continuous, fine-scale epidemiological monitoring is evident, as shown by a comprehensive cohort study in Northern Italy that provided insights into local HCV epidemiology [9]. This need for granular data is critical, as a systematic overview of the Middle East and North Africa highlighted the distinct regional prevalence patterns and transmission dynamics of HCV infection [10]. These regional and population variations underscore that successful HCV elimination requires not just high-level policy but also highly localized and efficient methods to accurately map disease burden and tailor treatment strategies to specific public health contexts, a challenge that remains critical worldwide.

Regarding treatment, the advent of direct-acting antivirals (DAAs) has markedly advanced HCV management [11]. Taiwan’s National Health Insurance (NHI) began reimbursing DAA therapy in 2017 for patients with advanced fibrosis (≥F3) or those unresponsive to interferon-based regimens [12]. By May 2018, over 9500 patients had received treatment, with a sustained virologic response at 12 weeks rate of 97.1% [13]. Since 2019, DAA coverage has expanded to all individuals with chronic HCV, irrespective of fibrosis stage, further aligning with national elimination goals. Collectively, these efforts contribute to Taiwan’s objective of eliminating HCV by 2030 [14].

Taiwan’s administrative hierarchy comprises city/county, township, and village levels. In our previous study, we established a township-level HCV risk map for Yunlin County, utilizing hospital-based surveillance data to guide screening in high-risk areas. That model was subsequently validated through community-based screening [15]. However, while previous official risk maps have primarily focused on the township level, emerging evidence increasingly suggests that HCV endemic foci may exist at even smaller, village-scale levels, necessitating refined, localized approaches [16].

Recognizing this critical research gap and the need for a more granular understanding, this study aimed to further investigate and alleviate the regional burden of HCV in Chiayi County. To support this national objective, our research specifically focused on two primary goals: first, to establish and validate a village-level risk model for HCV prevalence, and second, to implement a comprehensive community-based HCV screening and treatment initiative, specifically designed to identify village-level HCV hotspots, increase diagnosis rates, and facilitate local treatment.

## 2. Methods

### 2.1. Village-Level Mapping and Community-Based Screening of HCV Using Reflex HCV Antigen Testing in Chiayi County

This study was designed as a retrospective observational analysis combined with a community-based screening program. To identify high-prevalence areas of HCV in Chiayi County, village-level maps were developed for five endemic townships—Lioujiao (LJ), Yijhu (YH), Dongshih (DS), Taibao (TB), and Lucao (LC)—using data collected between 2000 and 2015 from the Chiayi Health Bureau and Chiayi Chang Gung Memorial Hospital. Specifically, the data combined public health screening campaigns conducted by the Chiayi Health Bureau with clinical records from the region’s only referral center, the Chiayi Chang Gung Memorial Hospital, thus providing a comprehensive surveillance picture of both community-based and clinical cases. Analysis of this database enabled the calculation of screening coverage and anti-HCV antibody prevalence, and facilitated the identification of screening-naïve populations. Among the five townships, only LJ and YH had previously undergone community screening [15]. In contrast, anti-HCV reflex Hepatitis C Virus antigen (Ag) testing was implemented in DS, TB, and LC to assess the village-specific prevalence of chronic HCV, ultimately designating these areas as targets for further investigation [17].

Building on the identification of high-prevalence villages, data integration between hospital and public health records was performed. This step precisely identified residents aged 30 years or older who had not been previously screened, thereby strengthening outreach and intervention efforts. All subsequent procedures were conducted after receiving approval from the Institutional Review Board (IRB) under protocols IRB No. 201802303B0, approval date: 5 February 2019 and IRB No. 202101844B0, approval date: 25 October 2021. Eligible individuals with at least six months of residency were invited to participate. Village-based outreach teams, composed of primary care personnel, conducted repeated door-to-door visits (each community was visited at least three times) to obtain informed consent and collect blood samples. Anti-HCV testing was performed, and reflex HCV Ag testing was conducted for those who tested positive, ensuring individuals confirmed to have active HCV infection were promptly referred for antiviral treatment.

### 2.2. Correlation of Pre-Screening and Post-Screening HCV Data

To assess the feasibility of using existing data to estimate community-level HCV prevalence, the study examined the correlation between prevalence estimates derived from pre-screening data and those obtained through actual community-based screenings in the five townships. It is hoped that these findings will aid in identifying and estimating remaining hidden HCV endemic areas in Taiwan, enabling targeted treatment and contributing to the ultimate goal of eliminating HCV.

### 2.3. Statistical Analysis

Geographic distribution and spatial patterns of anti-HCV prevalence were visualized using QGIS software (version 3.6). Furthermore, the number needed to test (NNT) is an epidemiological measure that represents the number of individuals who must be tested to identify one positive case of infection. In HCV screening, NNT provides a practical indicator of screening efficiency, with lower values reflecting higher prevalence and thus greater yield per test performed.

## 3. Results

### 3.1. Geographic Analysis Based on Pre-Screening Epidemiological Data on HCV

**Figure 1:**
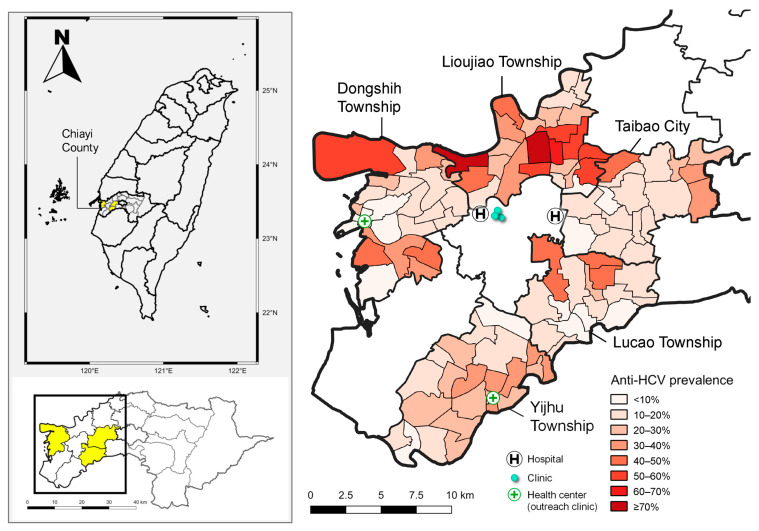
The map on the left illustrates the locations of Lioujiao Township, Yijhu Township, Dongshih Township, Taibao City, and Lucao Township in Chiayi County. The map on the right depicts the village-specific prevalence of hepatitis C virus antibodies along with the distribution of medical resources across the five townships. **Source:** National Open Data Platform, Taiwan. Township and district administrative boundaries (TWD97 latitude–longitude coordinates). Link: https://data.nat.gov.tw/dataset/7441 (accessed date: 21 November 2019).

In Figure 1, the map illustrates the anti-HCV prevalence across different townships in Chiayi County, Taiwan, using pre-screening epidemiological data on HCV derived from both hospital records and the Chiayi County Health Bureau.

The left map provides a geographic overview of Chiayi County in Taiwan, highlighting its location within the national and regional context. The right map offers a detailed, village-level visualization of anti-HCV prevalence across the five endemic townships: LJ, YH, DS, TB, and LC. This gradient map reveals a marked spatial heterogeneity in HCV exposure. Notably, several villages within DS, LJ, and TB exhibit very high anti-HCV prevalence, with some areas reaching ≥70% (Figure 1).

### 3.2. Pre-Screening Coverage Rate, Prevalence of HCV Ag and Anti-HCV

In Table 1, the pre-screening coverage rate of HCV varied across the three townships: DS, LC and TB. TB had the largest eligible population but the lowest prior screening rate (23.7%) and crude coverage (30.9%), indicating a substantial screening gap. LC, with the smallest population, achieved the highest prior (32.8%) and overall screening coverage (40.7%), reflecting more successful outreach or engagement.

### 3.3. Prevalence of Anti-HCV and HCV Ag, and Screening Efficiency (NNT) Based on Community-Based Screening

Based on the data presented in Table 2, our community-based outreach and screening strategy provided clear insights into HCV burden and the efficiency of case identification across three distinct areas. We first clarify the metric for efficiency: the number needed to test (NNT) represents the number of individuals who must be screened to identify one HCV antigen–positive case, with a lower NNT indicating higher screening efficiency. DS carried the highest disease burden, evidenced by the highest anti-HCV (8.7%) and HCV Ag prevalence (4.8%), which correlates directly with its finding of the lowest NNT (21), signifying the most effective case identification; furthermore, significant intra-township variation was observed in DS, strongly suggesting the presence of localized hotspots. In contrast, LC ranked second in screening efficiency with an NNT of 30, while TB exhibited the lowest prevalence rates (HCV Ag 2.4%) and the highest NNT (42), requiring 42 individuals to be tested to find a single HCV case. Overall, 132 individuals were identified as requiring treatment, and the follow-up demonstrated exceptional success in DAA treatment uptake: all cases in LC (31/31, 100%) and DS (57/57, 100%) received therapy, and 43 out of 44 cases (97.7%) were treated in TB, resulting in an overall treatment uptake of 131 out of 132 individuals (99.2%).

### 3.4. Geographic Analysis of Anti-HCV Prevalence Based on Community Screening and Village-Level Data Integration

**Figure 2:**
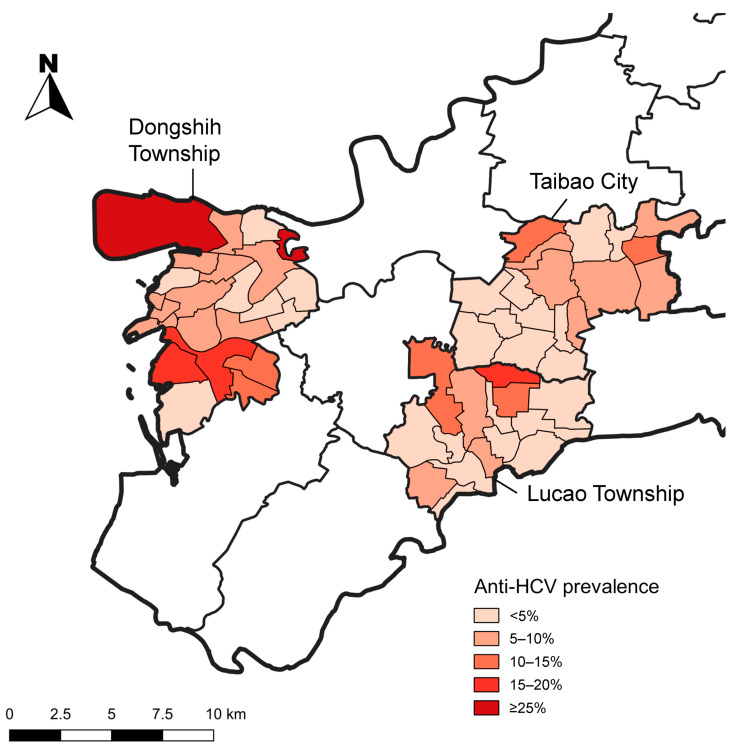
Geographic distribution of anti-HCV prevalence based on community screening and village-level data integration in Dongshih Township, Lucao Township and Taibao City. **Source:** National Open Data Platform, Taiwan. Township and district administrative boundaries (TWD97 latitude–longitude coordinates). Link: https://data.nat.gov.tw/dataset/7441 (accessed date: 21 November 2019).

In Figure 2, based on our study’s community screening and village-level data integration, this map illustrates the geographic distribution of anti-HCV prevalence across DS, LC and TB.

DS exhibits several areas with very high prevalence (darkest red, ≥25%), particularly in its northwestern and central-western regions. TB is shaded in red to orange, which places it among the higher-risk areas. In contrast, LC appears in light to medium orange, indicating a moderate burden of HCV.

### 3.5. Association Between Estimated and Observed Anti-HCV Prevalence at the Village Level

In the analysis of 56 villages across three townships, Pearson’s correlation coefficient between database-estimated anti-HCV prevalence and screening-based seropositivity was 0.696 (*p* < 0.001), indicating a moderate and statistically significant positive correlation.

When extended to 103 villages across five townships, the correlation further increased to 0.830 (*p* < 0.001), suggesting a very strong agreement between estimated and observed anti-HCV prevalence at the village level.

## 4. Discussion

This study demonstrates that a data-driven approach, utilizing pre-existing health records, can reliably and efficiently identify high-prevalence HCV hotspots. This method represents a significant advancement, as it allows for the precise estimation of disease burden at the village level, offering a more granular resolution than current official township-level risk maps. A pilot study has shown that conducting outreach HCV screening in rural and remote areas with high prevalence is safe and effective [18]. The successful application of this model, which showed a strong and statistically significant correlation between database-estimated prevalence and screening-based seropositivity (Pearson’s correlation coefficient of 0.830, *p* < 0.001), provides a valuable template for other regions with high HCV prevalence but limited data for guiding public health interventions [15].

To effectively eliminate HCV, minimizing patient dropout is significant, a challenge successfully addressed in this study. The exceptionally low patient dropout rate observed was primarily attributable to a streamlined, one-stop process that minimized both logistical and psychological barriers commonly faced by patients [19]. This success hinged on two key strategies. First, our “anti-HCV with reflex HCV Ag testing” strategy moved beyond the traditional two-step process, allowing us to conduct both screening and confirmatory testing from a single blood draw. While this approach significantly streamlines the workflow, it is important to note that HCV Ag detection is generally recognized as having lower sensitivity than nucleic acid amplification tests, such as HCV RNA, for confirming active infection. Nevertheless, this single-visit approach minimized multiple referrals—a common source of patient attrition in many screening programs [20]. Second, by delivering both screening and diagnostic results in one efficient workflow, we significantly reduced the waiting period and patient anxiety. This proactive approach ensured prompt linkage to care, keeping patients engaged and committed to the treatment pathway. Importantly, this strategy aligns with international recommendations, such as those from the European Association for the Study of the Liver (EASL), and has been adopted in other regions [21].

This highly successful mechanism for patient retention and engagement translated directly into an exceptionally high overall linkage-to-care rate, reaching 99.2% (131 out of 132 individuals), which significantly exceeds the WHO benchmark of 80% [3]. This remarkable treatment uptake can be attributed to several key factors that addressed major barriers to care. To establish accessibility, an outreach clinic was established in DS to provide local treatment in an area with limited healthcare resources. For residents in the other two townships, we ensured that treatment facilities were conveniently located within a 10 km radius (Figure 1), making DAA therapy easily accessible. This robust linkage-to-care mechanism successfully integrated community-based screening with accessible treatment services, as evidenced by the consistently high treatment uptake across all townships, including a 97.7% rate even in TB, the township with the lowest uptake (Table 2). Furthermore, this success was significantly supported by Taiwan’s National Health Insurance (NHI) policy. The NHI began reimbursing DAA therapy in 2017 and progressively expanded coverage to include all individuals with chronic HCV infection by 2019 [22,23]. This progressive expansion effectively eliminated the financial barrier to treatment, ensuring that virtually all eligible individuals were able to initiate and complete therapy.

The success of this comprehensive approach also underscores the critical value of our methodology: estimating HCV prevalence at the village level, rather than solely at the township level. This granularity is crucial for several compelling reasons, particularly for public health interventions. Our findings confirm that this village-level precision significantly increases efficiency for critical public health aspects, such as rational resource allocation, as relying on a single, averaged prevalence rate for a large area like a township is misleading and leads to inefficient strategies. This emphasis on using fine-scale geographic data to optimize resource deployment and intervention is consistent with similar spatial epidemiological approaches used in other settings to successfully identify and characterize HCV hotspots [24]. While the overall anti-HCV prevalence in DS was 8.7%, our detailed, village-level data revealed a stark reality: the prevalence within different villages of the same township ranged dramatically from 0% to a high of 29.4%. These significant intra-township variations are often attributed to highly localized factors, such as village-specific historical patterns of exposure (e.g., past use of non-sterile needles for medical procedures), unique micro-social determinants, and variations in community demographics and environmental factors [24]. By precisely identifying specific, highly endemic villages, public health resources—such as screening campaigns and mobile clinics—can be deployed exactly where they are needed most, optimizing impact rather than being spread thinly across an entire township.

Furthermore, estimating HCV prevalence at the village level significantly contributes to enhanced screening efficiency. Screening in these highly endemic villages proves far more efficient, a fact clearly demonstrated by the lower NNT in hotspots identified through our pre-screening model. For instance, screening in DS, which included high-prevalence areas, required an NNT of just 21, notably lower than the NNT of 42 observed in TB, a township with a lower overall prevalence. This targeted approach therefore maximizes the yield of positive cases per screening effort.

The success of this intervention validates the power of data-driven granularity. By precisely identifying HCV hotspots at the village level, we achieved significantly enhanced screening efficiency (NNT as low as 21 in endemic areas) and transformed public health outreach from general appeals into highly specific, persuasive calls to action. Crucially, the streamlined diagnostic process and robust treatment linkage resulted in an exceptional DAA treatment uptake of 99.2% (131/132). This scalable model demonstrates that targeted, prevalence-guided screening is the optimal strategy for maximizing resource efficiency and ensuring near-complete linkage-to-care.

## 5. Limitations

Despite these compelling findings, the study has several limitations. The reliance on historical data (2000–2015) for prevalence estimation, sourced from the Chiayi Health Bureau and Chiayi Chang Gung Memorial Hospital, may be influenced by population mobility. This is a plausible explanation for the observed discrepancy between the study’s findings and official HCV risk maps in TB, where the development of new residential areas has attracted a substantial influx of new residents. Additionally, the community-based approach may be subject to selection bias, as it primarily includes individuals who voluntarily participated in the screening. The challenge of the “registered but not residing” population, which limits the effectiveness of community-based campaigns, also underscores the need for broader, nationwide screening strategies to bridge this gap.

## 6. Conclusions

In conclusion, this study demonstrates that combining pre-existing health data with community-based screening enables the efficient identification of high-prevalence HCV areas at the village level. Our community-based interventions significantly increased screening coverage, while the treatment uptake exceeded 99%, highlighting a strong linkage to care. These findings support the use of local, data-driven approaches to guide targeted interventions and resource allocation, offering a model that can be applied to other high-burden regions to advance the 2030 WHO HCV elimination goals.

## Figures and Tables

**Table 1:** The demographics and screening coverage rate in Chiayi County.

Township	Taibao	Lucao	Dongshih
Population aged 30 years or older (A)	25,068	11,769	18,616
Ever been screened, *n* (%) (B)	5935 (23.7%)	3865 (32.8%)	4926 (26.5%)
Screening-naive (C = A − B)	19,133	7904	13,690
Participants of this screening (D)	1810	924	1176
Crude screening coverage rate (E = (B + D)/A)	30.9%	40.7%	32.8%

**Table 2:** The prevalence rates of antibody to hepatitis C virus, hepatitis C virus antigen and number needed to test in Taibao City, Lucao Township and Dongshih Township.

Township	Taibao	Lucao	Dongshih
Participants (*N*)	1810	924	1176
Number of villages	18	15	23
Prevalence of anti-HCV (*n*1, (%))	97 (5.4%)	56 (6.1%)	102 (8.7%)
Prevalence of HCV Ag (*n*2, (%))	44 (2.4%)	31 (3.4%)	57 (4.8%)
Rate of antigenemia (=*n*2/*n*1) (%)	45.4%	55.4%	55.9%
DAA treatment rate (DAA/HCV RNA+)	43/44 (97.7%)	57/57 (100%)	31/31 (100%)
Range of village-specific prevalence (%)	0~14.7%	0~15.9%	0~29.4%
Number needed to test (NNT) (*N*/*n*2)	42	30	21

## Data Availability

The data presented in this study are not publicly available due to institutional data governance restrictions and patient privacy concerns. However, the data may be accessed upon formal, written request and are subject to review by the Chiayi County Health Bureau and Chiayi Chang Gung Memorial Hospital.

## Abbreviations

The following abbreviations are used in this manuscript:

HCV	Chronic hepatitis C virus
LJ	Lioujiao
YH	Yijhu
DS	Dongshih
TB	Taibao
LC	Lucao
Ag	Antigen
NNT	Number needed to test
WHO	World Health Organization
DAAs	Direct-acting antivirals
NHI	Taiwan’s National Health Insurance
IRB	Institutional Review Board
EASL	European Association for the Study of the Liver
